# Deep Learning Algorithms-Based CT Images in Glucocorticoid Therapy in Asthma Children with Small Airway Obstruction

**DOI:** 10.1155/2021/5317403

**Published:** 2021-10-21

**Authors:** Yu Qin, Jing Wang, Yanjun Han, Ling Lu

**Affiliations:** ^1^Department of Pediatrics, Xingtai People's Hospital, Xingtai 054001, Hebei, China; ^2^Department of Endocrinology, Xingtai People's Hospital, Xingtai 054001, Hebei, China; ^3^Department of Pediatrics, Zaozhuang Municipal Hospital, Zaozhuang, Shandong 277100, China

## Abstract

CT image information data under deep learning algorithms was adopted to evaluate small airway function and analyze the clinical efficacy of different glucocorticoid administration ways in asthmatic children with small airway obstruction. The Res-NET in the deep learning algorithm was used to perform feature extraction, summary classification, and other reconstruction of CT images. A deep learning network model Mask-R-CNN was constructed to enhance the ability of image reconstruction. A total of 118 children hospitalized with acute exacerbation of asthma in the hospital were recruited. After acute exacerbation treatment, 96 children with asthma were screened out for small airway obstruction, which were divided into glucocorticoid aerosol inhalation group (group A, 32 cases), glucocorticoid combined with bronchodilator aerosol inhalation group (group B, 32 cases), and oral hormone therapy group (group C, 32 cases). Asthmatic children with small airway obstruction were screened after acute exacerbation treatment and were rolled into glucocorticoid aerosol inhalation group (group A), glucocorticoid combined with bronchodilators aerosol inhalation group (group B), and oral hormone therapy group (group C). Lung function indicators (maximal mid-expiratory flow (MMEF75 and 25), 50% forced expiratory flow (FEF50), and 75% forced expiratory flow (FEF75)), FeNO level, and airway inflammation indicators (IL-6, IL-35, and eosinophilic (EOS)) were compared before and one month after treatment. The ratio of airway wall thickness to outer diameter (T/D) and the percentage of airway wall area to total airway area (WA%) were measured by e-Health high-resolution CT (HRCT). The constructed network model was used to measure the patient's coronary artery plaque and blood vessel volume, and the image was reconstructed on the Res-Net network. It was found that the MSE value of the Res-Net network was the lowest, and the efficiency was very high during the training process. T/D and WA (%) of asthmatic children with small airway obstruction after treatment were significantly lower than those before treatment (*P* < 0.01). After treatment, MMEF75/25 and FEF75 were significantly higher than those before treatment (*P* < 0.05). Lung function-related indicator FEF50 was significantly higher than that before treatment (*P* < 0.01). FeNO level after treatment was remarkably lower than that before treatment (*P* < 0.01). In addition, lung function-related indicators, airway inflammation indicators, and FeNO level improved the most in group C, followed by group B, and those improvements in group A were the least obvious, with great differences among groups (*P* < 0.05). In summary, the Res-Net model proposed was of certain feasibility and effectiveness for CT image segmentation and can effectively improve the clinical evaluation of patient CT image information. Glucocorticoids could improve small airway function and airway inflammation in asthmatic children with small airway obstruction, and oral corticosteroids were more effective than aerosol inhalation therapy.

## 1. Introduction

Bronchial asthma, referred to as asthma, is a relatively common small airway disease, which is the chronic inflammation of the airway involving a variety of cells such as eosinophils and lymphocytes [1]. The incidence of asthma occurs in all ages, especially in children [2]. Studies have shown that children under the age of 14 suffer the most severe damage from asthma [3]. With the acceleration of urbanization and the increase of air pollution, the prevalence of childhood asthma in China is increasing year by year, which poses a serious threat to children's physical and mental health [4]. Glucocorticoids are the basic drugs for clinical treatment of asthma, which can not only participate in the multiple processes of airway inflammation, but also affect the permeability of capillaries. Moreover, it has a significant effect on alleviating bronchospasm and preventing pulmonary edema. As a result, the lung function is improved, and finally the onset of asthma is inhibited [5, 6]. There are many ways of drug treatment; systemic administration takes effect quickly, yet there are many adverse reactions; thus, it is restricted in clinical use. Local treatments are usually susceptible to drug particle size restrictions. However, the drug directly acts on the target organs, which is safe and effective. Studies have shown that glucocorticoid combined with bronchodilators can improve the clinical symptoms and pulmonary airway function of asthma patients, and the clinical efficacy is better than single administration. Whether the combination of drugs has the same effect on asthmatic children with small airway obstruction is still not clear. Therefore, exploring the different ways of administration of glucocorticoids is of great significance for the clinical treatment of asthmatic children with small airway obstruction.

The small airway is the most important part of asthma and chronic obstructive pulmonary disease. The onset of asthma is characterized by irreversible obstruction of the patient's airway. Therefore, small airways play a key role in the control and management of asthma diseases [7].

Medical image analysis is greatly influenced by subjective experience. The use of CT for auxiliary diagnosis of diseases can quickly and accurately judge diseases and reduce the rate of missed diagnosis and misdiagnosis in clinical diagnosis. It can improve the diagnosis rate of clinical lung cancer, thereby improving the quality of life of patients after surgery. The application of deep learning methods in images can assist physicians to make early disease diagnosis, positive treatment plans, and effective clinical decisions through measurement, evaluation, classification, diagnosis, and assisting preoperative design, which can effectively improve the efficiency of medical imaging for disease detection, recognition, and diagnosis and then promote and realize computer assisted treatment in the field of medical and health. Deep learning can realize image translation and other operations through weight sharing and other methods, optimize complex operations of image preprocessing, and achieve target detection and image classification [8]. With the rapid development of the e-health field, a variety of medical electronic products such as lung function instruments [9], spiral CT [10], and high-resolution CT (HRCT) [11] can be adopted in the detection of small airway diseases. Among them, high-resolution CT is the preferred method for evaluating small airway function, and it is also a hot spot in the field of imaging [12]. Dual-source computed tomography is a new device based on mature 64-slice CT technology. It has a great breakthrough in time resolution. The system imaging time resolution reaches 83 ms, which is 0.1 s less than the time required for cardiac imaging. The speed of each heartbeat is fast, which effectively improves the time resolution and has become the main method for noninvasive diagnosis of coronary heart disease. Dual-source CT has the characteristics of thin layer thickness, small pitch, small collimation, etc. The whole heart cycle exposure scanning method has a large radiation dose to the patient's blood vessels.

HRCT is of high sensitivity and specificity for the detection of small airway diseases, which can quickly collect the information of the lesion area and accurately identify the small structural changes of the airway. It is a safe, noninvasive, and easy-to-operate inspection method, which is widely used in clinical practice. Deep learning networks are often used in medicine to learn original images and are widely used in image segmentation, image classification, and target image positioning. Some scholars have proposed in research that combining pixel information of different scales can extract the best size information; some researchers believe that reducing the size of the convolution kernel can increase the running speed of the neural network. At present, there are few reports on the diagnosis of glucocorticoid in the treatment of children with asthma with small airway function obstruction by high-resolution CT image information data, and the study of deep learning method in this aspect is still insufficient. On the basis of the above research, this research further optimizes the convolutional neural network. The Res-Net network is used to reconstruct CT images, and a deep learning network model is constructed to enhance the ability of image reconstruction. By measuring lung function, airway inflammation indicator, and FeNO level, the clinical efficacy of glucocorticoid therapy was explored, to provide a theoretical basis for the assessment of asthma and the exploration of treatments.

## 2. Materials and Methods

### 2.1. Research Subjects

A total of 118 children who were hospitalized with acute exacerbation of asthma from October 2017 to October 2019 were selected, including 72 males and 46 females (6.86 ± 2.31 years). Inclusion criteria: I, patients aged 1–14 years; II, patients who met the diagnostic criteria for childhood asthma [13]; III, patients who could receive FeNO and lung function tests. Exclusion criteria: I, patients received glucocorticoid therapy in the past month; II, patients with antihistamine treatment history; III, patients with lung tissue fibrosis; IV, patients with pneumonia, chronic obstructive pulmonary disease, or malignant tumor; V, patients with poor compliance. This experiment had been approved by the ethics committee of the hospital, and the children and their families included in the study had signed an informed consent.

### 2.2. Grouping and Treatments

All patients received treatments of anti-inflammatory, anti-asthma, and phlegm, and the course of treatment was 7–12 days. Lung function indicators and FeNO levels were measured after symptom improvement. During this period, 2 children with respiratory failure were excluded. Then, according to the lung function test results, 8 patients with normal small airway function and 12 patients with obstructed atmospheric airway function were excluded. A total of 96 patients with small airway obstruction were screened, and the basic data of patients with mild obstruction (MMEF75/25 ≥ 50%) and patients with severe obstruction (MMEF75/25 < 50%) were compared.

Exclusion criteria for normal small airway function: FEV1/FVC > 70%, FEV1, FVC, and PEF were all greater than 80% of predicted values; there were no more than 2 indicators lower than 65% of predicted values among FEF50%, FEF75%, and MMEF75/25.

Exclusion criteria for atmospheric channel functional obstruction: FEV1/FVC < 70%, FEV1, FVC, and PEF were all less than 80% of predicted values.

Inclusion criteria for small airway obstruction: FEV1/FVC > 70%, FEV1, FVC, and PEF were all greater than 80% of predicted values; there were no less than 2 indicators lower than 65% of predicted values among FEF50%, FEF75%, and MMEF75/25.

A total of 96 small airway obstruction children were randomly divided into group A, group B, and group C. Patients in group A were treated with glucocorticoid aerosol inhalation (local administration), namely, budesonide suspension, twice per day. The children in group B were treated with glucocorticoid combined with bronchodilator aerosol inhalation (local administration), namely, budesonide suspension combined with terbutaline sulfate atomization solution, twice per day. Group C were treated with systemic drug administration, namely, oral methylprednisolone at 8 mg/day.

### 2.3. HRCT Detection

**Figure 1:**
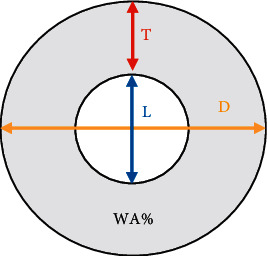
Schematic diagram of measurement of the inner and outer diameter of airway wall.

Patients were examined using Siemens Somatom Definition DSCT, Germany. The e-health equipment HRCT was adopted to examine asthmatic children with small airway obstruction. Target scanning technology was adopted, and scanning was performed when patients were peacefully breathing. The scanning range included the aortic arch, tracheal bifurcation and 2 cm below the bifurcation, 2 cm above the top of the pulmonary diaphragm, and the inferior pulmonary vein trunk, with a total of 5 layers. Scanning parameters: 120 kV, 200 mA; reconstruction parameters: 1200–1500 Hu window width, −500 window level, 3 mm layer thickness, and 2 mm layer spacing. Two or more experienced pediatric imaging physicians selected all levels of airway on the cross-sectional image for measurement. The measurement diagram of the airway wall inner diameter *L* and the airway wall outer diameter *D* is shown in Figure 1, and the average value was taken.

The ratio of airway wall thickness to outer diameter (T/D) was calculated according to the following equation, and the percentage of airway wall area accounting for airway cross-sectional area (WA%) was calculated via equation (2).

(1) $$\begin{array}{c} \frac{T}{D}=\frac{D-L}{2D}, \end{array}$$

(2) $$\begin{array}{c} \text{WA}\%=\frac{\pi {\left(D/2\right)}^{2}-\pi {\left(L/2\right)}^{2}}{\pi {\left(D/2\right)}^{2}}\times 100\%. \end{array}$$

### 2.4. Deep Learning Reconstruction Algorithm

**Figure 2:**
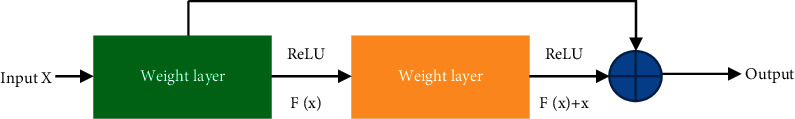
ResNet.

There are many algorithms for CT image reconstruction using deep learning, and the filtered backprojection algorithm can reconstruct CT images with better quality. The research on the network structure of deep learning is getting more and more in-depth, and the deep abstract semantic information of the image can also be extracted. As the number of layers increases, different neural networks have different trends in the classification of accuracy in the model. Networks such as AlexNet, GoogLeNet, and Res-Net are gradually decreasing in classification accuracy. The network depth keeps increasing, and the accuracy will increase to a certain extent, but beyond this level, there will be a gradient explosion problem, so the accuracy of the model training will decrease. In the NesNet network, there are residual errors and the results of the remaining modules, and its accuracy is better than that of networks such as AlexNet and GoogLeNet. As shown in Figure 2, the module connects the output to the input through skip connect. In the convolution calculation, a lot of image information corresponding to the input feature mapping of the residual module can be retained. This realizes that the NesNet network effectively improves the model when it is added in-depth.

### 2.5. Design of the Network Model

**Figure 3:**
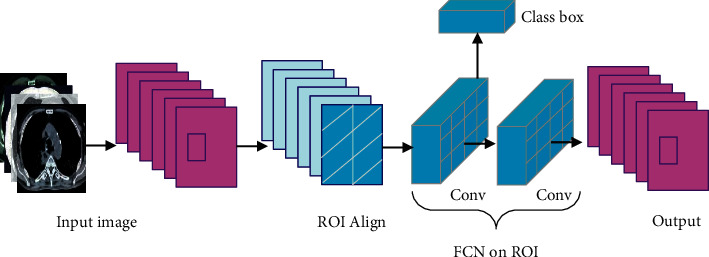
Structure of mask-R-CNN.

The R-CNN in the deep learning model follows the traditional target detection idea, first extracting candidate frames, and then extracting features for each frame. In the Fast-R-CNN network structure, classification, feature extraction, proposal extraction, and border regression are integrated into one network, which is conducive to the deep learning network to complete multiple tasks at the same time, and the detection speed is significantly improved. As shown in Figure 3, Mask-R-CNN adds a branch to Fast-R-CNN to obtain a new network framework. This new mask branch is applied to each Rol (Region of Interest). The small-scale fully convolutional neural network predicts the segmentation mask in a pixel-to-pixel manner, and a variety of flexible architecture designs are also added to the training. Mask-R-CNN uses bilinear interpolation to obtain the key coordinates of the small unit and performs the maximum pooling operation internally.

### 2.6. Model Improvement

The neural network trains the initial weight file on the MSCOCO data set in advance and trains it in the subsequent training process. The neural network of the multilayer residual block constitutes a semantic feature extraction module. The information after the module convolution operation and the input information are connected by the residual block through Skip connect to ensure the integrity of the information flow. The residual equation is as follows:

(3) $$\begin{array}{c} H\left(x\right)=F\left(x\right)+X. \end{array}$$

In equation (3), *F*(*x*) is the result of the convolution operation of the current residual block of the input information *X*. The upsampling function enables the input image to obtain deep abstract semantic information and restore feature maps of different sizes. When predicting the candidate frame, the feature map generated in the first step needs to have anchor boxes with different aspect ratios. If the size of the *i*-layer image is (*H*, *W*), and *n* is the number of candidate frame aspect ratios, then the aspect ratio *R* = [*R*1, *R*2, *R*3,…, *Rn*], *H* means height, and *W* means width; then, the equation for the total number of anchor boxes in this layer is expressed as

(4) $$\begin{array}{c} N=H\times W\times n. \end{array}$$

Anchor boxes of different specifications slide on the feature map, and the target area is not necessarily within the frame selection area. The selection of the candidate frame in the algorithm is based on the degree of overlap (IOU) between the content of the candidate frame and the gold standard. If C is the candidate frame area, and *D* is the gold standard area, then equation (4) indicates that the coincidence degree of the two areas is

(5) $$\begin{array}{c} \text{IOU}=\frac{\left(C\cap D\right)}{\left(C\cup D\right)}. \end{array}$$

Let *β* be the threshold; if IOU <*β*, the candidate region is considered as a positive sample; if IOU >*β*, the candidate region is considered as a negative sample. After the Anchor box detection is valid, the offset between the candidate box and the gold standard is calculated. Let the height and width of the candidate box be (H, W), the center coordinates of the Anchor box (*a*, *b*), the height and width of the image (*H*_1_, *W*_1_), and the target coordinates (*a*_1_, *b*_1_), and then the offset is

(6) $$\begin{array}{c} \Delta a=\frac{a_{1}-a}{W},\\ \Delta b=\frac{b_{1}-b}{h},\\ \Delta w=\log\frac{w_{1}}{W},\\ \Delta h=\frac{h_{1}}{h}. \end{array}$$

After the prediction of the candidate box, enter the Anchor box in the RPN network example for classification and regression. First, the gold standard corresponding to the positive sample and the screening are performed to obtain the score of the Anchor box. Calculate the cross entropy function. The cross entropy function can adjust the backpropagation and classification. The function equation is as follows:

(7) $$\begin{array}{c} {\text{LOSS}}_{C}=\sum_{j=1}^{T}y_{j}\log\frac{e^{aj}}{\sum_{K=1}^{T}e^{ak}}. \end{array}$$

In the equation, *y*_*j*_ represents the probability value of the gold standard corresponding to the positive sample, and *a*_*j*_ represents the predicted probability of the sample before normalization. The loss function calculation is performed according to the offset calculated when the anchor box is generated and the offset between the positive sample and the gold standard. The equation is as follows:

(8) $$\begin{array}{c} \text{smooth }L\left(x\right)=\left\{\begin{array}{cc} 0.5{\left(x\right)}^{2}, & if\left|x\right|＜1,\\ \left|x\right|-0.5, & \text{otherwise,} \end{array}\right.\\ L=\sum {\text{smooth}}_{L}\left(t-t^{*}\right). \end{array}$$

The target area scores are obtained for sorting, and the candidate frames with high scores are normalized by bilinear interpolation.

### 2.7. Detection Indicators

#### 2.7.1. FeNO

FeNO levels of children in each group were measured before and 1 month after treatment. Preparation before the test was as follows. Fasting food with high nitrogen content was conducted 10 hours before the test, and the consumption of stimulants drinks such as caffeine was prohibited. The strenuous activities were avoided, and the child was kept calm. Specific detection method: the nose clip was used to gently clip the patient's nose, and the filter was held by the mouth. Children breathed the air in lung out, then breathed normally, and kept the uniform speed of breath about 5 s.

#### 2.7.2. Pulmonary function

Pulmonary ventilatory function was measured before and 1 month after treatment in each group with the Jaeger Master Screen. Children should not take hormones, bronchodilators, and other drugs one day before the test. Two hours before the test, children should fast for solids and liquids, avoid strenuous exercise, and remain calm. Specific test method: children should sit and then close the nasal cavity and mouth with a nasal clip to completely contain the filter. After breathing normally several times, children should try their best to exhale the air in the lungs quickly. The test was repeated for several times, the three test values conforming to the quality control standard were taken, and the maximum value was taken as the final test result. The indicators of small airway were as follows: MMEF75/25, FEF50%, FEF75%, FEV1, PEF, and FVC. The improvement rate = (Posttreatment − pretreatment)/pretreatment × 100%.

Serum airway inflammation indicators: IL-35 and IL-6 levels were detected by ELISA, and EOS was determined by automatic blood count.

### 2.8. Statistical Analysis

SPSS22.0 statistical software was used for analysis. Normally distributed measurement data were expressed as mean plus or minus standard deviation ($\bar{x}$ ± *s*), and differences between groups were analyzed by variance analysis. If *P* < 0.05, the difference was statistically significant.

## 3. Results

### 3.1. Comparison of Clinical Data

A total of 118 children with bronchial asthma were treated in the acute exacerbation period, after which 12 cases of children with large airway injury were excluded, 8 cases of small airway function were normal, and 2 cases of respiratory failure were also excluded.  Table 1 shows comparison of basic data of children with different degrees of small airway obstruction. There was no significant difference in age, gender, and BMI between children with mild obstruction and children with severe obstruction (*P* > 0.05). The course of children with mild obstruction was 18.56 ± 8.69 (months), and the interval of medication was 7.36 ± 5.47 (h). The course of the children with severe obstruction was 20.26 ± 5.38 (months), and the interval of medication was 9.76 ± 6.59 (h). The duration of the disease in children with severe obstruction was longer than that in children with mild obstruction, and the time interval from onset to regular medication was longer than that of children with mild obstruction (*P* < 0.05).

Table 2 shows the comparison of baseline data of the three groups of asthmatic children with small airway obstruction. There were no statistically significant differences in age, gender, BMI, lung function-related indicators (MMEF75/25, FEF50, and FEF75), and FeNO levels between group A, group B, and group C (*P* > 0.05). However, the three groups had statistically significant differences in the course of disease and interval between medications (*P* < 0.05).

### 3.2. Imaging Characteristics of Asthmatic Children with Small Airway Obstruction

**Figure 4:**
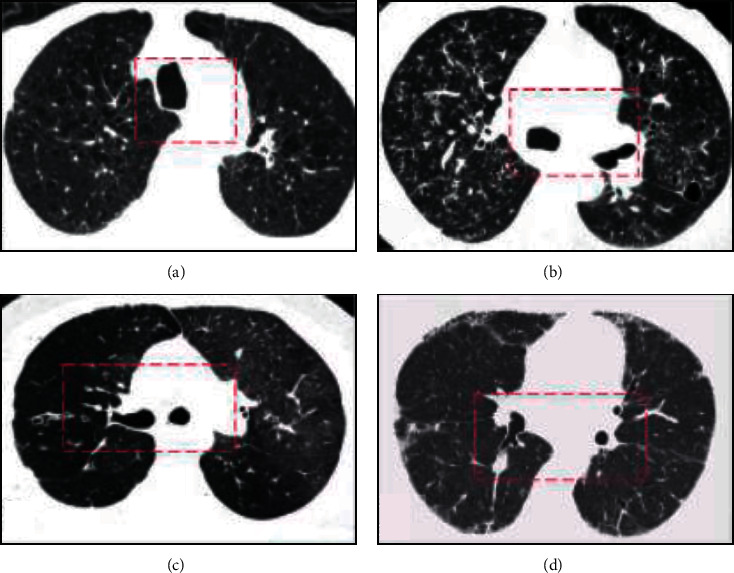
HRCT images of asthmatic children with small airway obstruction. (a) Bronchiolar wall thickening; (b) tree-in-bud; (c) air trapping; (d) mosaic sign.

The HRCT of e-health equipment was adopted to examine the characteristics of small airway lesions in asthmatic children with small airway obstruction, as shown in Figure 4. In some patients, the wall of the bronchioles had thickened, with small nodules, branch shadows, or ring shadows (Figure 4(a)). About 3–5 mm below the pleura, there were multiple small nodules and branch-like dense shadows distributed in the center of the leaflets, which looked like branch buds (Figure 4(b)). The lungs had limited low-density shadows and clear edges (Figure 4(c)). Irregular patterns or patches appeared in areas with increased lung density and areas with decreased lung density, but the edges were very clear (Figure 4(d)). The red box in the images indicated the location of the lesion.

### 3.3. Comparison of HRCT Test Results and Small Airway Function before and after Treatment

**Figure 5:**
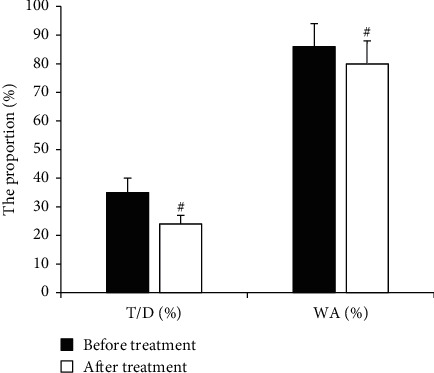
Comparison of T/D and WA (%) before and after treatment (^#^*P* < 0.01 as compared to that before treatment).

HRCT was used to detect changes in T/D and WA (%) in asthmatic children with small airway obstruction in asthma before and after treatment. In Figure 5, before treatment, T/D and WA (%) were 34.65 ± 3.86 (%) and 85.29 ± 12.54 (%), respectively. After one month of treatment, the T/D and WA (%) of asthmatic children with small airway obstruction were 23.38 ± 3.27 (%) and 78.65 ± 13.45 (%), respectively. The T/D and WA (%) of asthmatic children with small airway obstruction after treatment were significantly lower than those before treatment (*P* < 0.01).

**Figure 6:**
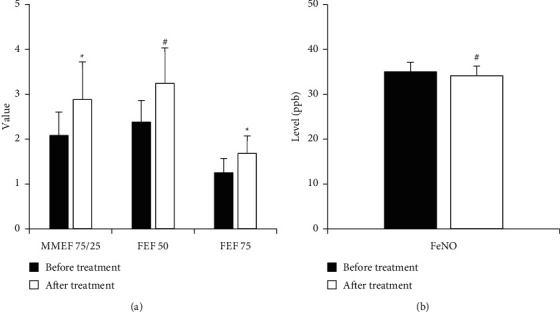
Comparison of lung function-related indicator and FeNO level before and after treatment. (a) Lung function-related indicator; (b) FeNO;  ^*∗*^*P* < 0.05 as compared to that before treatment; ^#^*P* < 0.01 as compared to that before treatment.

Before treatment, the MMEF75/25, FEF50, and FEF75 of asthmatic children with small airway obstruction were 2.08 ± 0.52, 2.38 ± 0.48, and 1.25 ± 0.32, respectively. After one month of treatment, the MMEF75/25, FEF50, and FEF75 of asthmatic children with small airway obstruction were 2.88 ± 0.84, 3.24 ± 0.79, and 1.68 ± 0.39, respectively. After treatment, the pulmonary function-related indicators MMEF75/25 and FEF75 were significantly higher than those before treatment (*P* < 0.05). After treatment, FEF50, a related indicator of lung function, was extremely significantly higher than that before treatment (*P* < 0.01) (Figure 6(a)). The FeNO level of asthmatic children with small airway obstruction was 35.00 ± 2.11 (ppb) before treatment and 34.12 ± 2.16 (ppb) after treatment. The FeNO level after treatment was lower than that before treatment (*P* < 0.01), as shown in Figure 6(b).

### 3.4. Comparison of Improvement of Small Airway Function in Each Group

**Figure 7:**
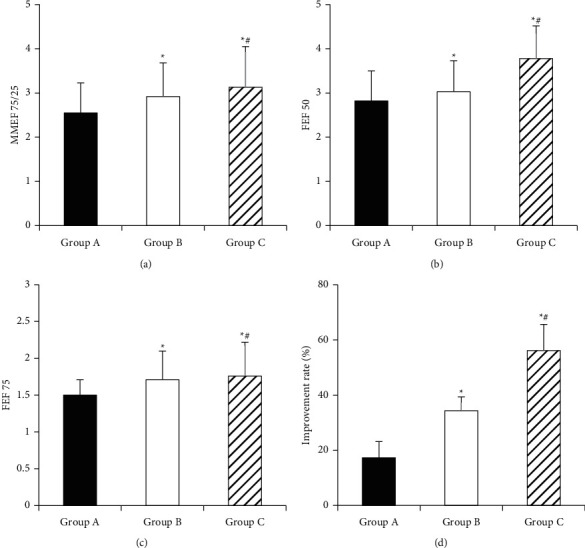
Comparison of lung function-related indicator after treatment in each group. (a) MMEF75/25; (b) FEF50; (c) FEF75; (d) improvement rate; ^*∗*^compared with group A *P* < 0.05; ^#^compared with group B *P* < 0.05).

Figure 7 shows the changes in lung function of children in each group after treatment. The MMEF75/25 values of group A, group B, and group C were 2.55 ± 0.68, 2.92 ± 0.76, and 3.13 ± 0.92, respectively; FEF50 values were 2.82 ± 0.68, 3.03 ± 0.70, and 3.78 ± 0.74, respectively; FEF75 values were 1.50 ± 0.21, 1.71 ± 0.39, and 1.76 ± 0.46, respectively. The improvement rates were 17.32 ± 5.87 (%), 34.36 ± 4.98 (%), and 56.12 ± 9.54 (%), respectively. The MMEF75/25, FEF50, and FEF75 values and improvement rate of children in group B were significantly higher than those in group A (*P* < 0.05). The values of MMEF75/25, FEF50, FEF75, and improvement rate of children in group C were significantly higher than those in groups A and B (*P* < 0.05).

**Figure 8:**
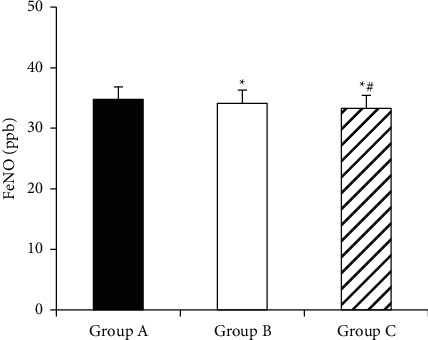
Comparison of FeNO levels after treatment in each group (^*∗*^compared to group A (*P* < 0.05; ^#^compared to group B *P* < 0.05).

Figure 8 shows the comparison of FeNO levels of children in each group after treatment. FeNO levels of group A, group B, and group C were 34.76 ± 2.07 (ppb), 34.12 ± 2.18 (ppb), and 33.28 ± 2.16 (ppb), respectively. The FeNO level of children in group B was lower than that of group A (*P* < 0.05), and the level of FeNO of children in group C was lower than that of groups A and B (*P* < 0.05).

### 3.5. Comparison of Airway Inflammation Indicators in Each Group

**Figure 9:**
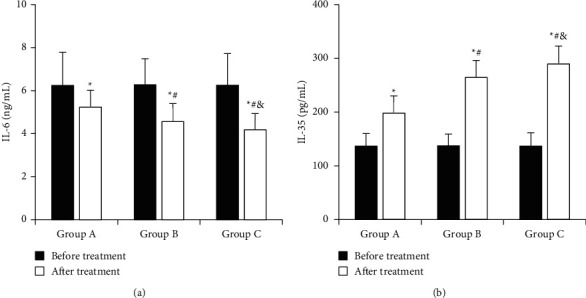
Comparison of IL-6 and IL-35 levels before and after treatment in each group. (a) IL-6; (b) IL-35; ^*∗*^*P* < 0.05, compared to that before treatment; ^#^*P* < 0.05, compared to that in group A; ^&^*P* < 0.05, compared to that in group B.

The comparison of IL-6 and IL-35 levels of children in each group before and after treatment is shown in Figure 9. In Figure 9(a), the IL-6 levels (ng/mL) before treatment in group A, group B, and group C were 6.24 ± 1.54, 6.27 ± 1.21, and 6.25 ± 1.47, respectively. The levels of IL-6 (ng/mL) after treatment were 5.23 ± 0.79, 4.56 ± 0.84, and 4.18 ± 0.75, respectively. After treatment, the IL-6 level of children in each group was significantly lower than that before treatment (*P* < 0.05), that of children in group B was lower than that of group A (*P* < 0.05), and that of children in group C was lower than that in group A and group B (*P* < 0.05). In Figure 7(b), the IL-35 levels (Pg/mL) of group A, group B, and group C before treatment were 136.67 ± 23.26, 137.56 ± 21.67, and 136.96 ± 24.72, respectively. The levels of IL-35 (Pg/mL) after treatment were 198.25 ± 32.02, 264.59 ± 31.64, and 289.65 ± 33.12, respectively. The levels of IL-35 in children in each group were significantly higher than those before treatment (*P* < 0.05), those in group B were higher than those in group A (*P* < 0.05), and those in group C were higher than those in group A and group B (*P* < 0.05).

**Figure 10:**
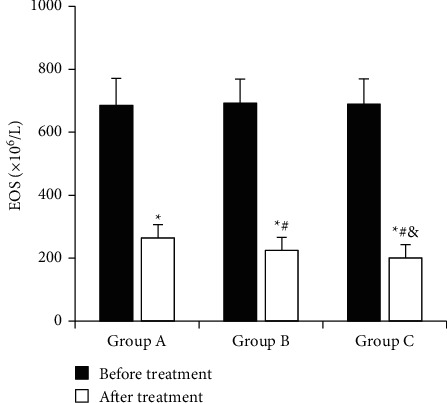
Comparison of EOS values before and after treatment in each group. ^*∗*^*P* < 0.05, compared to that before treatment; # *P* < 0.05, compared to that in group A; & *P* < 0.05, compared to that in group B.

The comparison of EOS values before and after treatment in each group is shown in Figure 10. The EOS (×10^6^/L) before treatment in group A, group B, and group C was 685.28 ± 86.26, 692.54 ± 76.78, and 689.62 ± 79.94, respectively. EOS (×10^6^/L) after treatment was 264.27 ± 42.65, 225.62 ± 40.37, and 201.35 ± 42.19, respectively. After treatment, the EOS of children in each group was significantly lower than that before treatment (*P* < 0.05), that of group B was lower than that of group A (*P* < 0.05), and that of group C was lower than that of group A and group B (*P* < 0.05).

### 3.6. MSE Curve

**Figure 11:**
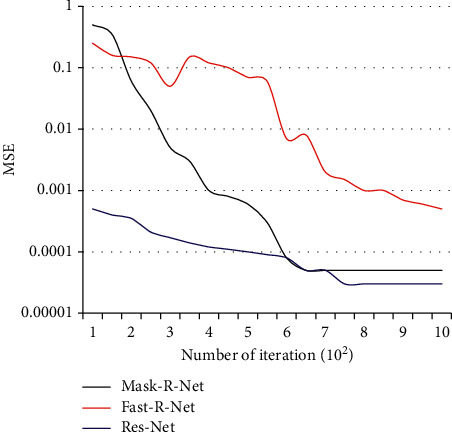
MSE curve diagram.

The smaller the MSE, the better the accuracy of the prediction model to describe the experimental data. In Figure 11, the Res-Net network has a smaller MSE than the other two networks, and the effect is extremely fast during the training process, indicating the improvement. The network effect is remarkable.

### 3.7. CT Image

**Figure 12:**
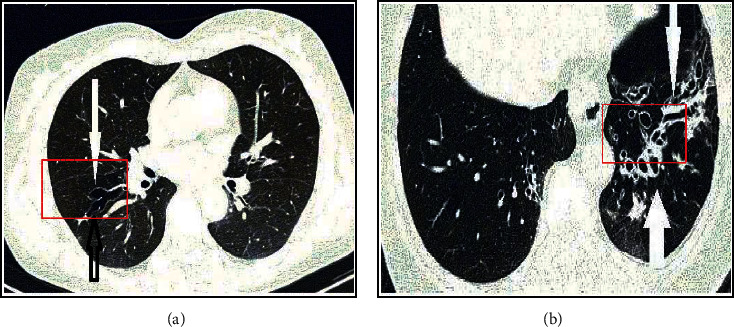
CT image of the patient's lungs.

Figure 12(b) shows a CT image of the lungs. The patient was a male 52-year-old patient. Compared with the right lung, the left lung is obviously branched. In Figure 12(a), the patient's lungs show that the bronchial tubes are not thinned but widened, which is a typical branch expansion. The red box in the figure shows the characteristics of the lesion CT.

## 4. Discussion

Asthma is a heterogeneous disease of the airways, whose attacks usually involve the air passages and can also cause obstruction or airflow limitation of small airways. e-health HRCT is an effective method to detect changes in airway structure. e-health HRCT was used to evaluate imaging changes in asthmatic children with small airway obstruction, and features such as thickening of bronchial wall, tree-in-bud, air trapping, and mosaic sign were found. It was almost consistent with the findings of Garcia-Clemente et al. [14]. In the research of Jiang et al. [15], 9 patients with small airway asthma and 20 healthy controls underwent HRCT with e-Health equipment, and the results showed that WA% in patients with small airway asthma was higher than that in healthy controls. In this research, T/D and WA (%) after glucocorticoid treatment were significantly lower than those before treatment (*P* < 0.01), which demonstrated the effectiveness of glucocorticoids in the treatment of asthma with small airway obstruction from the perspective of imaging.

FEEF50%, FEF75%, and MMEF75/25 are important clinical indicators for the evaluation of small airway function [16], and glucocorticoid is an effective method in preventing and treating asthma. FEEF50%, FEF75%, and MMEF75/25 were measured to evaluate the pulmonary function of asthmatic children with small airway obstruction before and after treatment. It was found that MMEF75/25 and FEF75 were significantly higher than those before treatment (*P* < 0.05), and FEF50 was significantly higher than that before treatment (*P* < 0.01). It suggested that, after glucocorticoid treatment, the pulmonary function of asthmatic children with small airway obstruction was effectively improved, which was consistent with the research results of Licari et al. [17]. FeNO is a marker of airway inflammation, and the greater the value, the more serious the airway obstruction [13]. It was found that FeNO level after treatment was lower than that before treatment (*P* < 0.01), indicating that glucocorticoid had alleviative effect on airway inflammation. Inflammatory cells in asthmatic children were activated, and there were more EOS in the airway epithelium, secreting a large amount of eosinophil basic protein, thus inducing the inflammatory damage of airway epithelium. The results showed that IL-6 and IL-35 played an important role in inflammatory response and airway hyperresponsiveness. It was found that IL-6, IL-35, and EOS of the children in each group were significantly improved after treatment compared with those before treatment, and the improvement was more obvious in group C compared with the other two groups. It suggested that glucocorticoids could relieve airway inflammation, and systemic administration was superior to local treatment. The results showed that aerosol therapy was superior to oral therapy. The atomizing device can disperse drugs into tiny droplets or particles, so that they are suspended in the gas, enter the respiratory tract or lung, and directly affect the drug on the lesion site, which can then wet the airway, clean the airway, and realize treatment effect.

Deep learning continues to develop rapidly. Researchers use repetitive programming of related programs to achieve effective deep learning algorithms. In deep learning, only the appropriate model needs to be selected, and the weight parameters of the model area are optimized after training. Chen et al. [18] simulated the evacuation design of buildings in the network model based on deep learning, introduced auxiliary image data prediction training algorithm, tracking sequence prediction training algorithm, and verified that the convolutional neural network model can predict data accuracy of the set. After the image is classified by deep learning, the location of the lesion can be displayed more clearly, and the characteristics of the lesion can be clearly classified. The image segmentation for deep learning in medicine includes convolutional neural network image segmentation and full convolutional neural network image segmentation. Frikel et al. [19] used microburst analysis to illustrate the appearance of artifacts in CT reconstruction images, which proved that deep learning algorithms can reconstruct the structure information of the scan target, and deep learning algorithms can achieve the effect of noise reduction.

## 5. Conclusion

The e-health equipment HRCT was adopted to diagnose childhood asthma with small airway obstruction. In this study, it was found that, after one month of treatment for children with asthma with small airway obstruction, the indexes related to lung function were significantly higher than those before treatment (*P* < 0.05), and the FeNO level of children with asthma with small airway obstruction was 35.00 ± 2.11 (ppb) before treatment and 34.12 ± 2.16 (ppb) after treatment. One month after treatment, T/D and WA were 23.38 ± 3.27 (%) and 78.65 ± 13.45 (%), respectively. Overall results showed that oral treatment was superior to aerosol inhalation. However, there are still some shortcomings in this research. For example, the number of samples is limited, and the clinic time and hospital are limited. Only the small airway function in one month after treatment is analyzed, but the long-term efficacy and possible side effects of the drug are not discussed. In the future, it needs to increase the diversity of sample regions and time, and further research on the effect of improving small airway function will be conducted. In short, the results of this study can provide a theoretical basis for the diagnosis and clinical treatment of asthma patients with small airway obstruction.

## Figures and Tables

**Table 1:** Basic conditions of children with different degrees small airway obstruction.

Item	Children with mild obstruction	Children with severe obstruction	F	*P*
Age (year)	6.38 ± 1.16	6.65 ± 0.98	0.103	0.747
Gender (male/female)	0.54 ± 0.52	0.49 ± 0.53	0.24	0.643
BMI (kg/m^2^)	24.01 ± 3.20	22.76 ± 3.37	0.421	0.527
Course (month)	18.56 ± 8.69	20.26 ± 5.38	3.853	0.049
Interval of medication (h)	7.36 ± 5.47	9.76 ± 6.59^*∗*^	9.763	0.001

*Note.* ^*∗*^The difference was significant.

**Table 2:** Basic data of asthmatic children with small airway obstruction in each group.

Item	Group A	Group B	Group C	F	*P*
Age (year)	6.43 ± 1.25	6.58 ± 1.05	6.63 ± 1.02	−2.650	0.674
Gender (male/female)	0.54 ± 0.52	0.49 ± 0.53	0.52 ± 0.50	0.421	0.643
BMI (kg/m^2^)	24.07 ± 3.24	23.97 ± 3.36	24.28 ± 3.12	0.088	0.765
Course (month)	21.37 ± 8.95	19.48 ± 9.88	20.64 ± 10.03	1.265	0.279
Interval of medication (h)	9.65 ± 6.91	6.97 ± 5.65	8.62 ± 7.84	0.786	0.426
MMEF75/25	2.12 ± 0.59	2.13 ± 0.48	1.97 ± 0.53	0.71	0.456
FEF50	2.36 ± 0.56	2.27 ± 0.43	2.40 ± 0.46	0.581	0.534
FEF75	1.28 ± 0.19	1.27 ± 0.26	1.18 ± 0.22	2.282	0.102
FeNO (ppb)	34.78 ± 1.98	35.01 ± 2.21	34.78 ± 2.04	0.289	0.732

## Data Availability

The data used to support the findings of this study are available from the corresponding author upon request.
